# Too Real to Be Virtual: Autonomic and EEG Responses to Extreme Stress Scenarios in Virtual Reality

**DOI:** 10.1155/2020/5758038

**Published:** 2020-03-12

**Authors:** Kirill A. Fadeev, Alexey S. Smirnov, Olga P. Zhigalova, Polina S. Bazhina, Alexey V. Tumialis, Kirill S. Golokhvast

**Affiliations:** ^1^Far Eastern Regional Scientific Centre of the Russian Academy of Education, Vladivostok, Russia; ^2^NTI Centre of Neurotechnologies and AR/VR Technologies, Far Eastern Federal University, Vladivostok, Russia; ^3^N.I. Vavilov All-Russian Institute of Plant Genetic Resources, Sankt-Peterburg, Russia

## Abstract

The evolution of virtual reality (VR) technologies requires setting boundaries of its use. In this study, 3 female participants were experiencing VR scenarios with stressful content and their activity of the autonomic nervous system and EEG were recorded. It has been discovered that virtual reality can evoke acute stress reactions accompanied by activation of the sympathetic nervous system and a decrease in the activity of the parasympathetic nervous system. The high-stress response is accompanied by a decrease in the power of the EEG, and, on the contrary, the activation of the avoidance reaction is accompanied by an increase in the power of the EEG alpha waves. Therefore, the use of stressful VR content can cause high emotional stress to a user and restrictions should be considered.

## 1. Introduction

Virtual reality (VR) is a computer-generated environment, where the user can freely navigate and interact with the virtual environment (VE) and its objects. Various virtual environments can be either representations of real places (for example, a virtual office space or a digital reconstruction of an ancient temple), or completely imagined, unrealistic (for example, when the user is in a place inaccessible to him, on the surface of another planet or inside a molecule). The widespread use of VR devices has begun in 2016 with the commercial release of HTC VIVE, Oculus Rift, Samsung VR, and PlayStation VR head-mounted displays (HMD).

The use of virtual reality has many cognitive and behavioral effects. However, the negative effects of experiencing some VR environments are also being noted. They can harm both the mental and physical health of the subjects; therefore, it is necessary to carefully choose the appropriate VR content. Various properties of virtual environments can have negative effects—the VR content induces or facilitates the reproduction of a negative or traumatic experience of the subject; the conflicts between vestibular, proprioceptive, and visual information trigger visually induced motion sickness (VIMS). The latter is a form of motion sickness caused by visual signals and, in the case of VR, is referred to as cybersickness.

How a person reacts and interacts to various virtual environments depends on a number of technological and psychological features, the most distinctive of which are immersion and sense of presence. The degree of immersion is determined by the technological characteristics of VR medium, such as HMD resolution, frame rate, field of view, stereoscopy, stereo sound, tracking accuracy, and speed. For instance, more photorealistic graphics in a virtual environment have a positive effect on immersion [1]. Another factor is the magnitude of sensory stimulation—the effect of immersion is greater in the case of simultaneous stimulation of more sensory systems, congruence of stimulations of different modalities, and its intensity [2]. The sense of presence (SoP) is defined as the subjective experience of being in one place or environment, even when one is physically situated in another. There is a relation between immersion and sense of presence. Cummings and Bailenson [3] have found that immersion has a medium-sized effect on presence. Some VR specifications, such as refresh rate and viewing angle, significantly affect the sense of presence. Additionally, increased levels of user tracking, the use of stereoscopic visuals, and wider fields of view of visual displays are significantly more impactful than improvements to most other immersive system features, including quality of visual and auditory content.

The ability to freely navigate through VE and interact with virtual objects, when their physics is realistically modelled, greatly contributes to the sense of presence [4, 5]. Stronger SoP is achieved when a user embodies his or her virtual avatar, which realistically depicts the user's movements with the help of motion capture technology [6]. The wording of the given instruction before the VE exposure [2] and the personality traits of the individual, such as the ability to immerse in various contexts, the ability to ignore distractions, the feeling of “being captivated,” the intensity of emotions provoked by the content, and the frequency of immersions into the content [7] also affect the strength of the sense of presence.

A subject's locomotion in a virtual environment evokes a side effect of VIMS, such as drowsiness, dizziness, pallor, cold sweat, oculomotor disturbances, nausea, and (rarely) vomiting [8]. In the virtual Roller Coaster simulation, VIMS scores had a positive correlation with the maximum nausea score during the ride and a negative correlation with the duration of the ride [1, 9]. However, the illusory sensations of own body movement (vection) and VIMS are not identical and have a complex indirect connection, because vection may occur without VIMS [10] and vice versa [11]. For example, Keshavarz et al. found that stimulus' density, speed, and rotations can alter the illusory sensations of self-motion and have additive effects, which is not associated with the VIMS [10]. Keshavarz et al. suggested in 2019 that vection is a necessary prerequisite, but not a sufficient condition, for inducing VIMS. Moreover, in certain conditions, the effect of SoP in VR has a positive correlation with vection [10], but is negatively associated with VIMS [12].

Three hypotheses have been put forward regarding the nature of VIMS [8]. The first and the most common is the theory of sensory conflict between visual, vestibular, and somatosensory information. The second theory postulates that VIMS arises as a result of individual instability in controlling posture. The third is the hypothesis of eye movements, stating that the moving visual pattern causes optokinetic nystagmus, which is associated with the activation of n. vagus, leading to VIMS.

In addition to subjective assessments of the psychological state, registration of subjects' physiological reactions also showed significant changes when using VR. Free movement in a VE increases the number of blinks and the amplitude of EDA, heart rate, and tachygastric power, and decreases bradygastric power, respiratory sinus arrhythmia, finger temperature, and respiratory rate [13, 14]. Similar effects of sympathetic activation were found in the virtual Roller Coaster [15, 16] or simulated in VR high places [17, 18].

The EEG studies have found various results. In the earliest study [13], in the VE navigation task, the spectral power of the delta rhythm increased and the spectral power of the beta rhythm decreased. Also, controlled navigation in VR, compared to uncontrolled navigation, caused an increase in the presence and desynchronization of the power of alpha and theta rhythms in the right insula and the alpha rhythm in the right parietal region [19]. A large desynchronization of the alpha rhythm in the parietal cortex was accompanied by a decrease in coherence with the frontal region, interpreted by the authors as a decrease in inhibitory control of the frontal cortex with a higher sense of presence [20]. Compared to the control condition for the horizontal movement of the cart, the Roller Coaster caused a decrease in the alpha rhythm power in the middle and posterior cingulate cortex and upper parietal cortex in adolescents [15]. A study using evoked EEG potentials showed that the subjects with a high reported state of presence in a VE had a lower amplitude of late EP components for a rare target stimulus, which, according to the authors, was associated with a focus on the content of the VR scenario [20].

Studies using tomography have shown that the extent of presence in the VE negatively correlated with the activity of the lateral frontal cortex. Moreover, a decrease in the presence effect is associated with a decrease in the activation in the thalamus and egocentric dorsal visual processing stream as well as upregulating medial areas of the PFC, involved in self-reflective thoughts [21]. In another study [22], free navigation in the VE is associated with the activation of the posterior parietal cortex and the insula. In the Roller Coaster VE, hemodynamics in the parietal-temporal region of the head, measured by NIRS, also increased [9]. Thus, the activity of the parietal cortex, as well as the lateral frontal cortex and the insula, is associated with the effect of presence and navigation in a VE. But there is little research on this topic, and the conclusions therefore are more likely to be hypothetical than affirmative.

Due to the negative effects on the emotional and physical state of the subjects, it is necessary to follow strict ethical requirements, and it is also difficult to attract subjects to participate in such study. Investigations of pre-, peri-, and posttraumatic processes in the VR paradigm have revealed that VR content induces traumatic stress [23], and they also showed that stressful VR content causes mild stress symptoms, such as those related to trauma thoughts and beliefs, intrusion, and avoidance behavior [24, 25]. The assumption that people with OCD, PTSD, and psychotic disorders are more vulnerable to cybersickness has previously been put forward, but when tested, conflicting results were received. Some researchers found a positive effect after using VR for the diagnosis and treatment of psychotic disorders [26], while others did not [27]. In relation to this study, exposure to a moving field of vision in patients with unilateral labyrinthopathy led to nausea earlier and more often than in a group of healthy people and less than in the group with bilateral labyrinthopathy [28], which may make this group vulnerable to cybersickness. Also, this group likely includes people with postural instability, so a study of healthy people with individual differences in spontaneous postural instability showed greater vulnerability to cybersickness [29]. However, there are no studies on the side effects of VR on users with vestibulopathy, as there are no studies on the safety of VR experiences to persons with certain diseases, and, accordingly, there are no recommendations. The reviews in [8, 12] indicate that in studies using VR, weak VIMS is caused, or the subjects with severe symptoms of VIMS are removed from the analysis. This is noted as a significant lack of research, and therefore, the involvement of a specific group of subjects exposed to the negative effects of VR with high and pronounced emotional reactions can contribute to a more accurate diagnosis of symptoms.

In the current study, subjects were selected according to the criterion of the presence of strong negative emotional and physiological reactions to specific VR content, which contrasts with the general tendency to exclude such subjects from studies. Due to the limited number of such subjects in the population, their low motivation to repeat negative experiences during the experiment, and the existence of ethical standards that require minimizing damage to the subjects, in this study we obtained data from only three female volunteers. We investigated their strong negative emotional reactions in specific VR scenarios: a Roller Coaster and an interactive tour outside the International Space Station. As control conditions, the subjects were registered at a rest state and also were presented with the city view VE. In addition, the second group of control conditions included VR environments such as a flight over the city and the outer space, which served to control the conditions of open space and the lack of support underfoot. High frame rate, stereoscopy, realism, high speed, and acceleration of movement were to contribute to the increase of immersion and to the appearance of VIMS symptoms in the mentioned stressful scenarios.

Thus, the aim of this study was to study physiological responses to specific stressful VR scenarios in subjects with abnormally intense emotional responses to VR scenarios that may cause VIMS.

## 2. Methods

Authors confirm that all experiments on humans were performed in accordance with relevant guidelines and regulations of the Ethic Committee of the Far Eastern Federal University in 2018.

### 2.1. Participants

The subjects, who complained of an acute emotional reaction during introductory sessions using VR from February to April 2019, were interviewed about their negative experiences with VR, and then they were asked to repeat the unpleasant VR experience in the experiment. The participation was voluntary; all the participants were informed in detail about the nature of the VR scenarios and signed an informed consent. All participant were older than 18 years old.

Three female subjects participated in this study.

Subject #1 (20 years old) regularly visits a neurologist because a year ago she was diagnosed with vestibulopathy, radiculopathy C5-C6 and C6-C7, subluxation C5-C6 and C6-C7, and vertebral artery syndrome. According to her, she had a birth injury of the upper neck. She is experiencing persistent general headaches, occasional vertigo, frequent nosebleeds, and a feeling of pulsation in the head since birth. Since the effect of the pain medication prescribed by the neurologist is negligible, the subject does not take the prescribed medication.

Subject #2 (34 years old) has chronic cerebral ischemia, vestibuloatactic syndrome, and cephalgic syndrome as a consequence of ischemic stroke in the vertebrobasilar basin in 2013. She is experiencing regular migraines, dizziness, decreased attention, and reactions. She receives postischemic treatment in the day hospital every 6 months, and for some time she took prescribed antihypoxants, nootropics, and drugs to improve cerebral circulation, such as Mexidol, Cerebrolysin, Actovegin, Phenibut, and Vasobral. However, at the time of participation in the experiment, the subject did not take these drugs for more than 8 months.

Subject #3 (20 years old) has a sports trauma. She visited a traumatologist, a neurologist, and a therapist and other specialists and was diagnosed with cervical vertebra fissure in the upper neck and a pinched nerve. She experiences frequent dizziness, and when she bows her head to the left there is also a numbness in the left hand.

### 2.2. Materials and Methods

All experimental protocols were approved by the Ethics Committee of the Far Eastern Federal University (confirmed by the Ethics Committee conclusion of April 19, 2019).

The subjects wore the HTC VIVE VR HMD (https://www.vive.com). This HMD is well established in the VR consumer market, and it enables high-fidelity graphics with a display resolution of 1080 × 1200 for each eye (2160 × 1200 combined pixels), 90 Hz refresh rate, a 110° field-of-view, and a precise infrared tracking. The interaction with the VR environment takes place through the included HTC VIVE controllers. The headset was connected to a PC with an Intel Core i7-7700, 16 GB RAM, NVIDIA GeForce 1080, and 220 GB SSD.

EEG recording was performed using the NVX136 EEG amplifier (https://mks.ru/product/neurovisor-136/) and the MCScap (https://mcscap.ru/) with a set of 60 passive Ag/AgCl electrodes placed according to the 10-10 system. The AFz channel was used for grounding, and the reference electrodes were set on the participant's mastoids. The high-pass filter was 0.05 Hz, and the low-pass filter was 50 Hz. The sampling rate was 1000 Hz. The ECG, the EDA, and the respiratory rate were recorded as well. For ECG recording, disposable Ag/AgCl electrodes were placed on the participant's forearms. The online filter was set to 1-40 Hz. Ag/AgCl electrodes located on the second phalanxes of the index and ring fingers of the left hand were used to register EDA in the skin conduction variant. The online filter was set to 0.05-10 Hz. Due to technical issues, EDA was recorded only on Subject #3. The respiratory rate was recorded with a nasal thermal sensor, and the filter was set to 0.05-10 Hz.

### 2.3. VR Scenarios

Emotional reactions were evoked within free and paid VR applications, available through Steam or Oculus Home services:

1. Richie's Plank Experience (https://store.steampowered.com/app/517160/Richies_Plank_Experience/) is a casual VR game, where the user appears on a busy sidewalk in front of an elevator of an 80-story skyscraper. There, the user may continue to stay on the ground or he/she may enter the elevator and take it to the upper levels with different gameplay options: on the 2nd level there is a fire extinguisher to put down a fire on the terrace; on the 3rd level the player is allowed to fly freely through the city with a VIVE controller acting like an engine with a built-in smoke generator; and on the last elevator button, the player is brought to the level with a wooden plank, 80 stories high over the street. The developers of Richie's Plank Experience position this product not as a game, but rather as a psychological experience to test one's ability to withstand the evoked fear of height. The game has high ratings on Steam (83% of 380 users' reviews are positive as of 24/07/2019). The ground level of Richie's Plank Experience was used as a neutral or control condition, and the 3rd level was used as a “Flight” scenario. The subjects were asked to do a free flight over the city. They had control with a VIVE controller over the height and speed of the flight. Both scenarios from this application lasted for 4 mins each
2. Epic Roller Coasters (https://store.steampowered.com/app/787790/Epic_Roller_Coasters/) is a casual VR game available with a free trial. The user rating for this game is moderate (58% of 209 users' reviews are positive as of 24/07/2019), and many of the users have noted that this virtual roller coaster ride experience is very similar to a real one. This VR scenario was proposed as stressful and lasted for approximately 4 min. During the ride, subjects were seated and could not control the speed of the cart
3. Nature Treks VR (https://store.steampowered.com/app/587580/Nature_Treks_VR/) is a paid interactive VR walk simulator which allows the user to visit various virtual environments, similar to a real one, like a tropical beach or a rainforest, or completely surrealistic, like, the birth of the Universe in the endless vacuum of nothing. The game has a very positive rating among users who bought it (86% of 175 users' reviews are positive as for 24/07/2019). Its latter level was used in this study as a Cosmos scenario and was characterized by the lack of floor, borders, or other visual cues. The subjects were seated and their avatars just floated in the endless space filled with stars for 4 minutes.
4. Home—A VR Spacewalk (https://store.steampowered.com/app/512270/Home__A_VR_Spacewalk/) is an interactive VR tour on the International Space Station (ISS) made by BBC and available for free. The player is on a guided mission to exit the ISS through the hatch and inspect solar panels, when the high-velocity space debris hits the space station and the situation suddenly changes into the worse. Thrown away from ISS during the impact with his safety line cut, air leaking, and visor broken, the player has to orient in the open space and fly back to the hatch using the Manned Maneuvering Unit. Duration of the tour is approximately 10-15 min. The application has realistic graphics and zero gravity physics, and it received good reviews from players (74% of 161 users' reviews are positive as of 24/07/2019) and utilizes the VIVE controllers to grab numerous ISS's handrails and interact with space things.

Due to technical issues, the Cosmos scenario has not been presented to Subject #2. All the scenarios, except the ISS one, lasted for 4 mins. The ISS scenario lasted 10 mins for Subject #1, 15 mins for Subject #2, and 12.5 mins for Subject #3.

### 2.4. Procedure

Subjects arrived at the laboratory, and after the EEG cap and other sensors were connected, the registration took place for 4 mins with open eyes and for 2 mins with closed eyes (rest). Then, the HTC VIVE HMD were put on, and VR scenarios were presented in the following order: a ground level of Richie's Plank Experience (City), a 3rd level of the same game (Flight), Epic Roller Coasters free-fall level (Roller Coaster), Nature Treks VR The Black Beginning level (Cosmos), and the ISS tour. There were 3-5 min breaks between scenarios for subjects to rest and for experimenters to prepare the next scenario. If the subject experienced a too high emotional reaction, she could signal the laboratory staff to stop the scenario at any time.

### 2.5. Analysis

Analysis of psychophysiological reactions was carried out for 4 min segments from each VR scenario, except the ISS, where two 4 min segments were chosen to distinguish the less stressful first part of the tour, where the player has to open the hatch and travel outside the station by grabbing handrails (ISS1), and the second part, when the dramatic accident occurs and the player finds himself in open space detached from the ISS (ISS2).

EEG data was imported into EEGLAB 14.1.1 (https://sccn.ucsd.edu/eeglab/). The 1-20 Hz filter was applied. A low cutoff filter of 20 Hz was chosen to remove myographic artifacts caused by subjective reactions to VR scenarios (squinting, wrinkling and other facial movements, general muscle tension, etc.) and by VR hardware (HMD and environmental electromagnetic noise). Subject's #2 T8 electrode was deleted and then restored using interpolation. After this, EEG data was rereferenced to the common average, and independent components were calculated using the Infomax algorithm. The removal of components containing artifacts occurred semiautomatically. Components with severe oculographic and myographic artifacts have been removed. Electrooculography was not performed because the VR HMD was placed around the eyes. EOG artifacts were deleted based on the standard topographic profiles of the individual components and the distinctive temporal pattern of these artifacts.

Furthermore, the data were examined for the presence of residual artifacts, and then the spectral power of the EEG segments with a duration of 4 seconds with an overlap of 2 seconds was calculated using Fast Fourier Transform. The Pz electrode was used to analyze the spectral power of the alpha rhythm, and the frequency limits of the alpha rhythm ranged from 8 to 13 Hz; when the FCz electrode was used for the analysis of the spectral power of the FM theta rhythm, the frequency limits of the rhythm were from 4 to 7 Hz.

EDA data was imported into the Ledalab 3.4.9 package (http://www.ledalab.de/). First, the data was downsampled to 10 Hz, then the Butterworth filter order 1 with a low cutoff frequency of 5 Hz was applied, and finally, the number and average amplitude of spontaneous fluctuations of EDA above the threshold of 0.01 *μ*S were calculated.

ECG data was imported into the Kubios HRV 2.2 (https://www.kubios.com/), and HR, RMSSD, lnLF, lnHF, and correlation dimensions were analyzed.

The breathing data were analyzed using standard MATLAB functions. The breathing peaks were extracted, and the average breathing rate was calculated.

## 3. Results

### 3.1. Subject #1

All the VR scenarios before Roller Coaster were performed without pronounced emotional reactions. After the Roller Coaster, she felt a moderate motion sickness, so the recovery period lasted about 6 minutes. The next VR scenario (Cosmos) was done calmly. After 8 mins in the ISS, she stopped the scenario, being in a pronounced negative emotional state. The subject describes her condition as follows: “I was calm until I needed to go out into outer space. When the meteorite storm started on the ISS, I felt a loss of support under my legs, the sensation of approaching loss of consciousness, a loss of balance, dizziness. Then I closed my eyes, took off the headset and cried, I wanted to stop the experiment immediately, I felt a headache, a throb in my head and a veil before my eyes.” Some symptoms, such as dizziness, persisted until one week. In this case, we witnessed an episode of paroxysmal anxiety. These paroxysms of pronounced anxiety are limited to the situation caused by the ISS scenario. It took about one hour to restore Subject #1's emotional state with the assistance of a psychologist. Based on this, we assume that we observed the debut of a phobic disorder, a predictor of which was vestibulopathy.

#### 3.1.1. Respiration Rate

The mean breathing rate was lower at rest and in the City scenario while it was at a maximum in the Flight scenario. Then, it gradually decreased in the Roller Coaster and Cosmos scenarios and increased again in both periods of the ISS scenario (Table 1).

#### 3.1.2. Heart Rate

**Figure 1:**
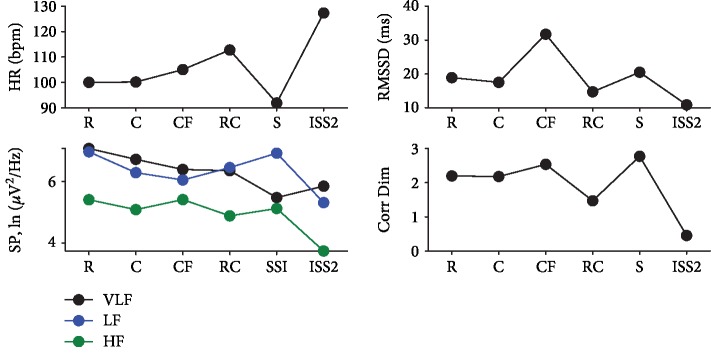
Heart rate variability results of participant 1. List of scenarios: R—rest with eyes open; C—city; CF—flying above the city; RC—roller coaster; S—space; ISS2—second period of analysis within the International Space Station scenario; heart rate variability indices: VLF—very low frequency; LF—low frequency; HF—high frequency; HR—heart rate in beats per second; SP—spectral power; Corr Dim—correlation dimension.

In the first period of the ISS scenario, there were a lot of motor artifacts, which made it impossible to analyze the data. HR in the rest state and in the Cosmos scenario was high, but then it has been linearly increasing in the Roller Coaster and ISS2 VEs. The lowest HR was in the Cosmos scene. Root mean square of successive heart rate interval differences (RMSSD) was maximum in the Flight scenario and minimum in the ISS2 period relative to the Rest level. Very low frequency (VLF) gradually decreased across scenarios. Low-frequency (LF) and high-frequency (HF) changes were slight from the first scenario in space and were lowest in ISS2. The correlation dimension was the smallest in the ISS scenario and the largest in the Cosmos scenario (Figure 1).

Thus, according to the heart rate data, a decrease in parasympathetic activity was detected in the ISS scenario, which possibly caused an increase in heart rate. Also, the Roller Coaster scenario showed a similar tendency but of a smaller amplitude. The Roller Coaster and ISS scenarios also caused an increased breathing frequency; however, in the case of the Cosmos scenario, we observed an opposite breathing response, i.e., relaxing.

The EEG spectral power in the FCz in the theta rhythm frequency band (4-7 Hz, FM theta) did not have a pronounced pattern of reaction to the scenarios.

**Figure 2:**
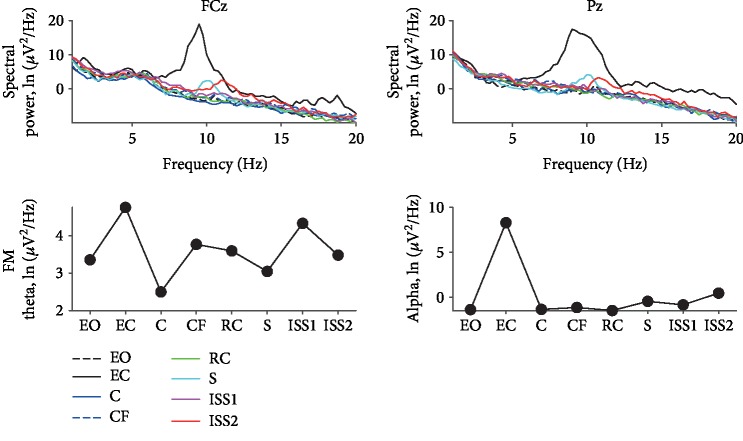
EEG spectral power results of participant 1. List of scenarios: EO—rest with eyes open; EC—rest with eyes closed; C—city; CF—flying above the city; RC—roller coaster; S—space; ISS2—second period of analysis within the International Space Station scenario.

The EEG spectral power in the Pz in the alpha rhythm frequency band (8–13 Hz) has a maximum spectral power in the closed-eyes condition. Across scenarios, Subject #1 shows a gradual increase in spectral power, and in the second period of the ISS has the maximum power, but significantly less than in the closed-eyes condition (Figure 2).

### 3.2. Subject #2

According to the subject: “I was very scared on the Roller Coaster, they were fake, but the sensations that I would fall out were real. It seemed to me that I felt how the body moves in space with the cart, in the most terrible moments I tried to tell myself that all this is not real, to calm down. I rested against the back of the chair and the floor with my feet to feel that I was still there, and not on a Roller Coaster. My palms were sweating, but my body was cold, (I felt) dizziness, tingling in my limbs, shortness of breath, feeling of a rush of blood to my head, palpitations.” According to her subjective assessment, the most stressful scenario was a Roller Coaster due to acrophobia, while the ISS and Flying were less stressful.

#### 3.2.1. Respiration Rate

The respiration rate was lower in the rest condition, in the City scenario, and in the first period of the ISS scenario as compared with the scenarios of flying over the city, Roller Coaster, and the second part of the ISS scenario (Table 1).

#### 3.2.2. Heart Rate

**Figure 3:**
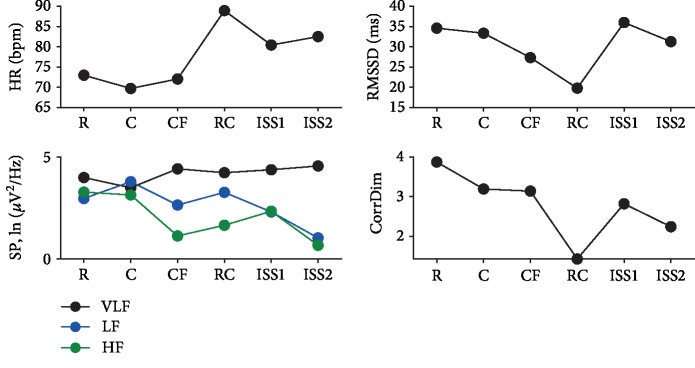
Heart rate variability results of participant 2. List of scenarios: R—rest with eyes open; C—city; CF—flying above the city; RC—roller coaster; ISS2—second period of analysis within the International Space Station scenario; heart rate variability indices: VLF—very low frequency; LF—low frequency; HF—high frequency; HR—heart rate in beats per second; SP—spectral power; Corr Dim—correlation dimension.

The HR in the Roller Coaster scenario rose and remained elevated in the ISS scenarios, relative to the Rest, City, and Flight scenarios. The RMSSD reflects the parasympathetic activity, which is significantly reduced in the Roller Coaster scenario compared to the rest state and the City scenario. Also, there were lower values in the Flight scenario and ISS2. The correlation dimension was also significantly reduced in the Roller Coaster scenario. The VLF power remained stable in all scenarios. The LF power was also stable from Rest to ISS1 and had the lowest values in ISS2 relative to Rest. The values of HF were smaller in the Flight scenario, ISS2, and Roller Coaster scenario compared to the Rest state (Figure 3).

Therefore, the Roller Coaster scenario had the strongest impact on Subject 2, both subjectively and in terms of the reactivity of her vegetative system. Flying over the city and in the International Space Station scenarios were less stressful. All of these scenarios reflected the subject's fear of heights.

**Figure 4:**
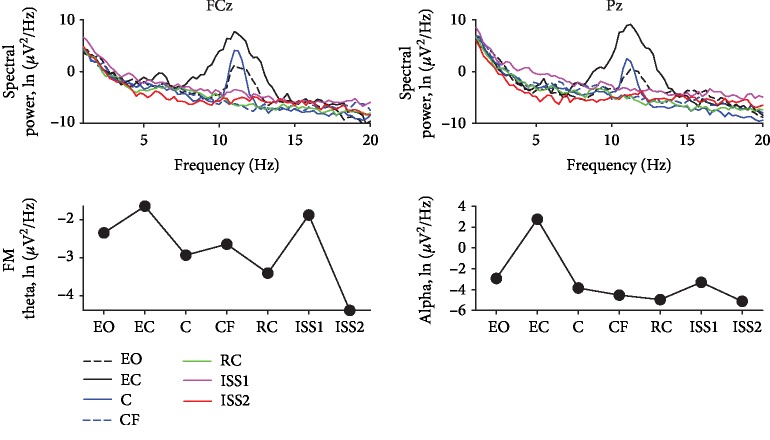
EEG spectral power results of participant 2. List of scenarios: EO—rest with eyes open; EC—rest with eyes closed; C—city; CF—flying above the city; RC—roller coaster; ISS1—first period of analysis within the International Space Station scenario; ISS2—second period of analysis within the International Space Station scenario.

The spectral powers of the FM theta and parietal alpha rhythm tended to decrease the power in stressful Roller Coaster and ISS2 scenarios, reflecting an increase in the level of central activation (Figure 4).

### 3.3. Subject #3

The subject noted that the stress level in the Roller Coaster scenario was the highest, and the ISS scenario was less stressful. According to the subject, during the Roller Coaster, she experienced the feeling of dizziness, instability, fainting, slight nausea, and difficulty breathing. However, the subject, despite the stress, decided not to stop the experiment. In addition, she was feeling dizzy for several hours after the Roller Coaster scenario.

#### 3.3.1. Respiration Rate

The breathing rate increased gradually in the City and the Flight scenarios compared to rest. In the Roller Coaster scenario, the breathing rate was the lowest and increased gradually in the Cosmos scenario and in the first and second periods of the ISS scenario (Table 1).

#### 3.3.2. Heart Rate

**Figure 5:**
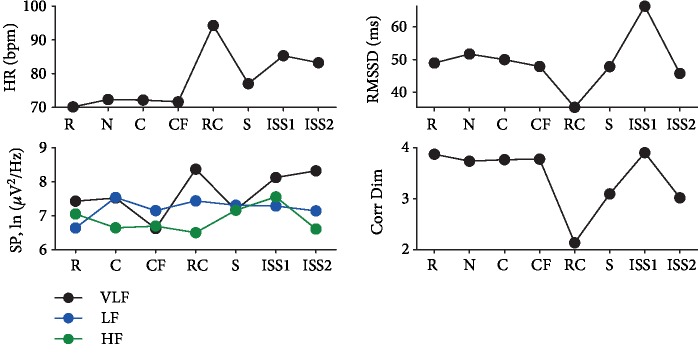
Heart rate variability results of participant 3. List of scenarios: R—rest with eyes open; C—city; CF—flying above the city; RC—roller coaster; S—space; ISS1—first period of analysis within the International Space Station scenario; ISS2—second period of analysis within the International Space Station scenario; heart rate variability indices: VLF—very low frequency; LF—low frequency; HF—high frequency; HR—heart rate in beats per second; SP—spectral power; Corr Dim—correlation dimension.

Relative to the Rest condition, the heart rate increased in the Roller Coaster scenario and remained elevated in the ISS scenario. RMSSD and correlation dimension were reduced in the Roller Coaster scenario. The spectral power values are varied slightly (Figure 5).

### 3.4. Electrodermal Activity

There were no spontaneous fluctuations exceeding the threshold in the Rest condition, the City, and the Flight over the city scenarios. In the Roller Coaster scenario, 24 fluctuations with a mean amplitude of 0.035 *μ*S were found. In the Cosmos scenario, there was a single fluctuation with an amplitude of 0.012 *μ*S. In the first period, there were 15 fluctuations with a mean amplitude of 0.045 *μ*S and there were 5 fluctuations with a mean amplitude of 0.038 *μ*S in the second period of the ISS scenario.

Thus, the results of vegetative activity indicate that the increase in sympathetic activity (in terms of the number and mean amplitude of EDA fluctuations) and the decrease in parasympathetic activity (in terms of RMSSD and lnHF) were in the Roller Coaster and, to a lesser extent, in the ISS scenarios.

### 3.5. EEG Spectral Power

**Figure 6:**
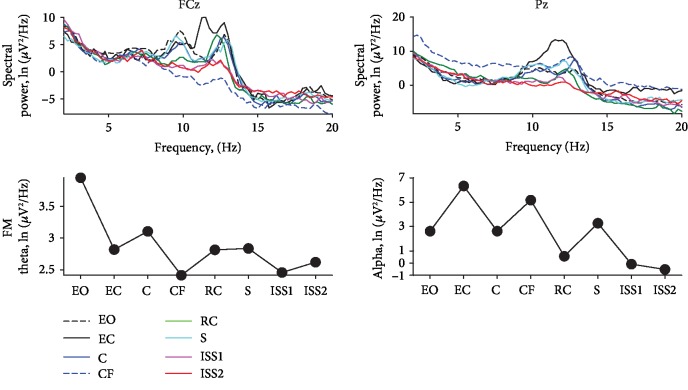
EEG spectral power results of participant 3. List of scenarios: EO—rest with eyes open; EC—rest with eyes closed; C—city; CF—flying above the city; RC—roller coaster; S—space; ISS1—first period of analysis within the International Space Station scenario; ISS2—second period of analysis within the International Space Station scenario.

The alpha rhythm spectral power was low in the Roller Coaster and ISS scenarios. The power of the FM theta rhythm was greatest in the rest condition with open eyes and then remained the same across all conditions (Figure 6).

Thus, the level of activation measured by the desynchronization of the alpha rhythm was most pronounced in the stress scenarios of the Roller Coaster and the ISS.

## 4. Discussion

The study investigated the negative emotional reactions to stressful VR scenarios in three female subjects. The results showed a decrease of the parasympathetic and an increase of the sympathetic autonomic nervous system activities, as well as a less specific response of the EEG spectral power in the theta and alpha bands.

Virtual reality evoked emotional reactions through the effects of immersion and the presence in VR environments. Previous studies have shown that in moderate-intensity stress, VR scenarios evoked a sympathetic activity [15–18]. In the present study, these data were confirmed; the current results showed that electrodermal amplitude and the number of fluctuations were increased in the most stressful ISS scenario in one subject, reflecting the increase in sympathetic activity. The most stressful VR scenarios for each subject in the current study were also accompanied by a decrease in the high-frequency heart rate variability, suggesting a decrease in parasympathetic activity. Studies [30, 31] showed a positive effect of high-frequency heart rate variability on the control of the emotional response; on the contrary, its decline is induced by stressful situations [32], confirming the present results and suggesting the uncontrolled emotional response to stressful scenarios. Correlation dimension was never used before for the analysis of autonomic reactivity in stressful VR scenarios. The current result shows a decrease in HR dimensionality in stress, strongly differentiates the stressful scenarios and rest, and suggests that this is the good measure of stress reaction.

In studies, conflicting results were obtained regarding changes in the respiratory rate during navigation in VE. In the study [14], the respiratory rate increased on average by more than 30% relative to the rest condition in the task of free navigation in VE. In the study by Kim et al. [13], the respiratory rate fell by about 20% in the task of finding objects in VE. In the present study, the respiratory rate increased in the flight scenario by approximately 24%; in the first and second periods, the ISS increases were by 6.1% and 10.3%, respectively. The increase in effects in the second period of the analysis of the ISS scenario relative to the first period of analysis can reflect both the more stressful situation and the cumulative effect of VIMS during a stay in a VE [14]. In the Roller Coaster scenario, the respiratory rates of first and second subjects increased by 20.6% and 24.2%, respectively, and decreased in the third subject by 14.3%. The raw data inspection revealed that the third subject has a greater breathing period in the Roller Coaster due to the breath holding in most stressful ride periods. Two other subjects showed higher respiration rates than the average values, according to [14], during their stay in the VR scenario. Also, all three subjects had higher respiration rates at rest, compared with the data [13, 14]. These differences may be due to the fact that in this study, the subjects were aware of the upcoming test and might have formed expectations. An increase in the respiratory rate is also associated with an increase in sympathetic activity [33]; therefore, it can be assumed that the subjects developed an expectation of an aversive state, but nevertheless responded to stressful situations with a significant increase in the respiratory rate. In more stressful scenarios, the respiratory rate tended to increase, although there were also individual differences. It should also be taken into account that the greatest increase in the respiratory rate occurred in the flight scenario, which caused weak emotional reactions.

Together, the increase in sympathetic and the decrease in parasympathetic activities resulted in an increased heart rate in stressful scenarios compared to control scenarios. In [9, 14, 32], the average heart rate reactivity was 5–8 beats per second; in the study [1], subjects with high subjective estimates of nausea had an increase in heart rate of 13 beats per second. In studies [1, 9], an increase in heart rate had positive relationships with nausea, which also had negative relationships with the length of stay in a stressful environment. In the current study, subjects had an increase in heart rate in stressful scenarios relative to rest at about 20 beats per second. Thus, these data suggest that the scenarios in this study were highly stressful for subjects. Self-reports also indicate that, after stressful episodes, subjects experienced acute stress reactions, accompanied by cold hands and tremor, and cold sweat, although data were not recorded objectively. The emotional effect lasted for several days.

The VR scenarios of the second group of control conditions, the Flight and Space, were used to control the increase of the viewing space and elevation above the level of the ground, and reduce the perception of support. These scenarios showed results similar to scenarios of the first control group, i.e., Rest state and the City. Thus, autonomic activity, as well as subjective state, suggested that the increase of visible space and the decrease in perception of support did not evoke intensive emotional reactions. Also, the flight scenario consisted of moving over the city and evoked vection and more likely positive than negative emotional reactions. These results are consistent with data [8] on the complex relationship between vection and VIMS. Finally, the absence of effects of emotional and autonomous activation in the second group of control conditions and the presence of pronounced reactions in stress scenarios associated with significantly more intense spatial movements suggest that under stressful conditions, subjects experienced an acute episode of cybersickness, detected only in acute stressful episodes. An alternative hypothesis is that flying over the city is a less realistic scenario, which caused a decrease of the effect of presence. In contrast, a stationary and more realistic plank at a height causes emotional and autonomous reactions [17, 18].

Brain activity, according to the EEG spectral power, showed mixed results. There is a general effect of reducing the power of the alpha rhythm in the rest condition with open eyes and in all VR scenarios compared to the rest condition with closed eyes. This effect, as well as the presence of blinks in the scenarios when processing EEG, suggests that during the VR scenarios, the subjects did not close their eyes. However, across all scenarios for the first subject, the power of the alpha rhythm in the most stressful scenarios increases, while for the second and the third subjects, the power drops. It should be noted that the first subject stopped the most stressful ISS scenario, while the second and third subjects did not. Perhaps, the increase in the power of the alpha rhythm reflects the activity of the mechanisms of sensory inhibition in the most stressful situation, and it is also indicative of the actualization of avoidance motivation. Studies showed that an increase of the alpha rhythm power is associated with an increase in sensory thresholds [34, 35] and is in inward association with the focus of attention [36, 37]. On the contrary, two subjects in the most stressful situations showed a decrease in the power of the alpha rhythm drawing attention to sensory analysis [38], perhaps reflecting the presence of reserve to control their emotional state. The data from these two subjects correspond to the results of previous studies, showing alpha power desynchronization in the parietal area in scenarios with navigation in a virtual environment [15, 19, 20] due to the activation of the parietal cortex [21, 22].

The power of the FM theta rhythm reflect the activity of cognitive control mechanisms [39] and should impact in brain reaction to the stressful scenarios. However, the present data did not show pronounced changes in FM theta power associated with the stress magnitude.

Thus, the study of three subjects showed that the acute emotional stress reactions were evoked by specific VR scenarios. In particular, highly intense subjective and autonomic reactions to stressful scenarios associated with fast and uncontrolled movement in a VE indicate a high degree of negative emotional activation in subjects with vestibulopathy and fear of heights. Dysfunction of the vestibular apparatus is a predictor of some phobic (panic) disorders, such as agoraphobia and acrophobia [40–42]. Panic attacks result from dissociation between objective and subjective assessments of the risk of falling, which was observed in Subject #1 with vestibulopathy. Therefore, the psychological safety of such content for users with vestibulopathy and phobias is in question. The research community and national governments should develop recommendations for the use of VR applications, like the Motion Picture Association of America film rating system.

The results may be of interest to the clinicians dealing with emotional disorders, such as PTSD or panic disorder and also for the developers of VR applications. Virtual reality technologies are evolving and becoming more accessible to consumers. Together with the positive effects, consideration should be given to the limitations of their use.

## Figures and Tables

**Table 1:** Breathing rate (breaths per minute).

Subject	Rest	City	Flight	Roller Coaster	Cosmos	ISS1	ISS2
1	15.4	14.6	23	19.4	15.4	19.4	17.6
2	18.2	18.8	24	24	—	18.8	21.4
3	17.6	18.8	20.7	15.4	15.8	16.7	18.2

## Data Availability

Data available upon request.
